# Supplementary material for: PEPPRO: quality control and processing of nascent RNA profiling data
Source: Genome Biol. 2021 May 15;22:155. doi: 10.1186/s13059-021-02349-4 (PMC8126160; doi:10.1186/s13059-021-02349-4)
Supplement: Supplementary file 1 — Additional file 1 Example R code to generate a gene counts table and supplemental figures. [file 13059_2021_2349_MOESM1_ESM.pdf]

Additional File 1

R code to generate a gene counts table

PEPPRO provides a project level counts table that simplifies downstream analyses. Here, we import the PEPPRO project counts table and construct a DESeq data set in a few lines of code.

```
# 1. Load the PEPPRO R package.
library(PEPPROr)

# 2. Load the PEP project using the project configuration file.
prj = Project("peppro_paper.yaml")

# 3. Load the project gene counts table.
counts = read.csv(file.path(paste0(config(prj)$metadata$output_dir,
                            "/summary/PEPPRO_countData.csv")))

# 4. Only keep the H9 untreated or H9 HDAC inhibitor treated samples.
counts = counts[,c("geneName", "H9_PRO-seq_1", "H9_PRO-seq_2", "H9_PRO-seq_3",
                   "H9_treated_PRO-seq_1", "H9_treated_PRO-seq_2",
                   "H9_treated_PRO-seq_3")]

# 5. Convert the counts table to a matrix by removing the gene name column.
count_matrix = as.matrix(counts[,-"geneName"])

# 6. Set the rownames of the matrix object to be the gene names.
rownames(count_matrix) = counts$geneName

# 7. Create a data.frame that defines the sample information.
coldata = data.frame(condition=c(rep("untreated", 3), rep("treated", 3)))

# 8. Set the rownames of the sample information data.frame to match the counts matrix.
rownames(coldata) = colnames(count_matrix)

# 9. Load the DESeq2 package.
library("DESeq2")

# 10. Create a DESeq data set from our counts matrix and the sample information data.frame.
dds = DESeqDataSetFromMatrix(countData = count_matrix,
                             colData = coldata,
                             design = ~ condition)
```

Supplemental figures

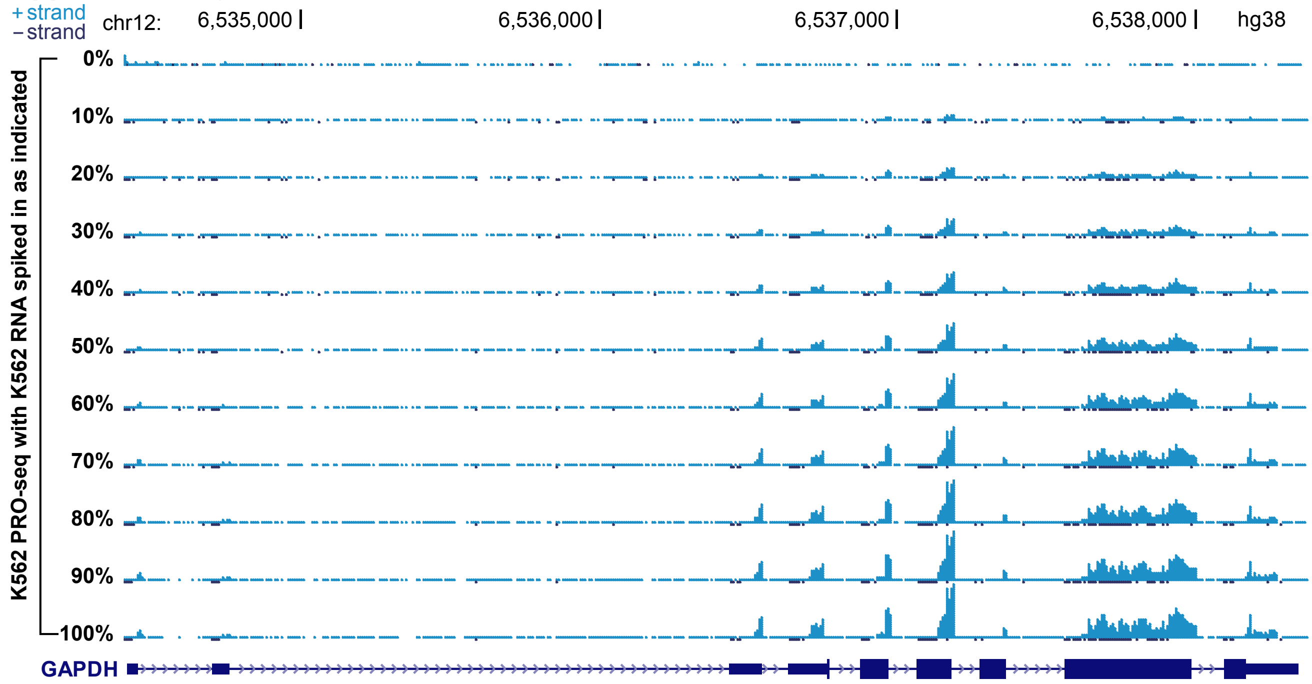

*Fig. S1: **K562 RNA-seq spike-in signal tracks show increasing exonic coverage**. GAPDH exonic coverage is enriched as the percentage of RNA-seq reads increases, and is visualized particularly well at exons 6 and 8. Each sample library is composed of 70M total reads. Scale for each track is 1000 to -20.*

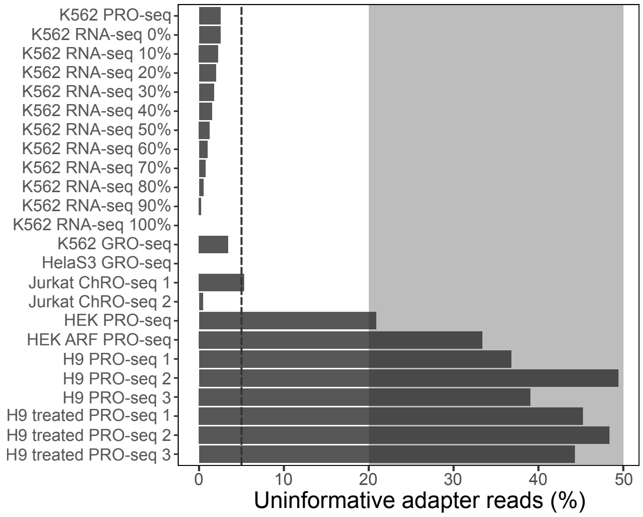

*Fig. S2: **Percentage of uninformative adapter reads following adapter removal for test set samples**. The HEK and H9 libraries contain more adapter-adapter reads because PAGE-mediated size selection was excluded from the protocol (Values below the dashed line are generally recommended for PAGE-purified libraries. Shaded region represents the recommended abundance of adapter reads for libraries* without *PAGE purification.)*

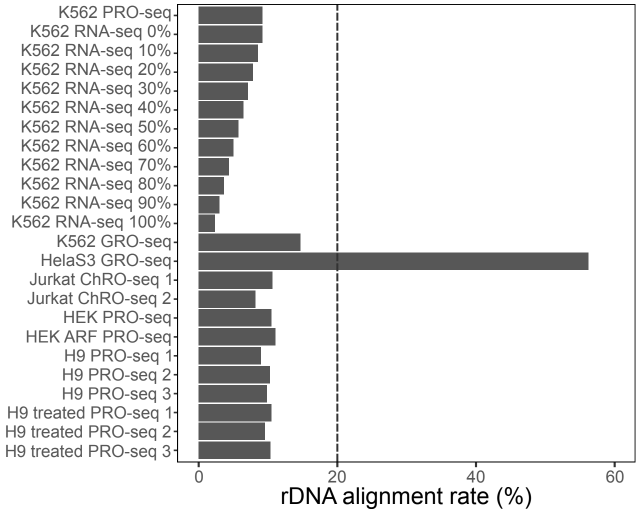

Fig. S3*:* ***Ribosomal DNA alignment rates for test set samples****. The HelaS3 GRO-seq sample is highly enriched for ribosomal RNA transcripts compared to other test samples. Values below the dashed line are recommended.*

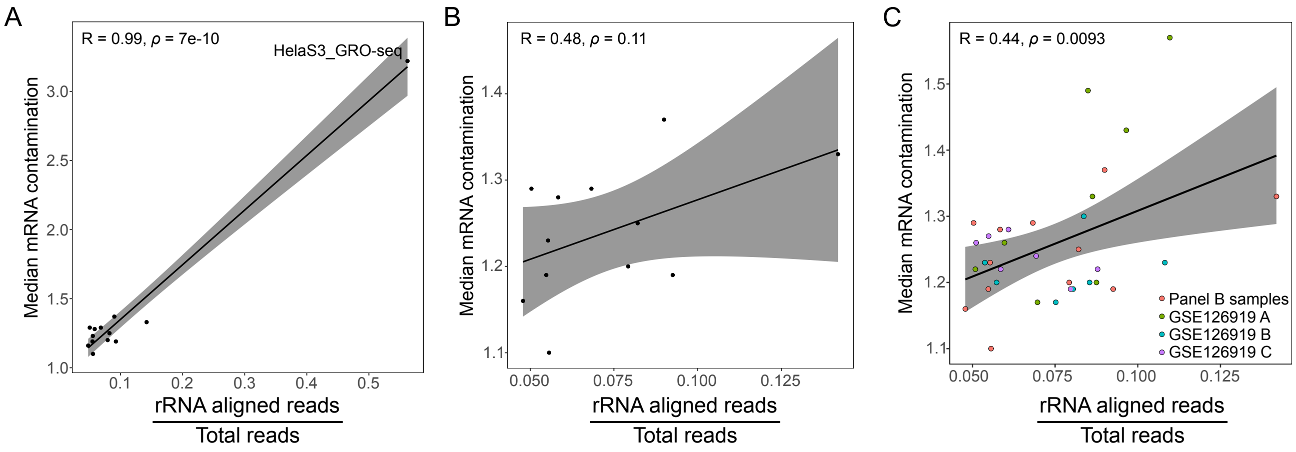

Fig. S4*:* ***Abundance of rDNA to total reads is correlated with mature RNA contamination.*** *Correlation plot between the measure of mRNA contamination (median exon:intron density) and the ratio of rDNA aligned reads to total reads for: A) all primary samples (*excludes RNA-seq spike-in experiment due to ribosomal depletion inherent in RNA-seq library preparation), B) primary samples excluding the known outlier HelaS3_GRO-seq sample, C) all samples in panel B* and *all non-redundant samples from GSE126919 including three cellular subclones (A, B, and C) to demonstrate possible differences due to cell lines. Test for association determined with Pearson's product moment correlation coefficient.*

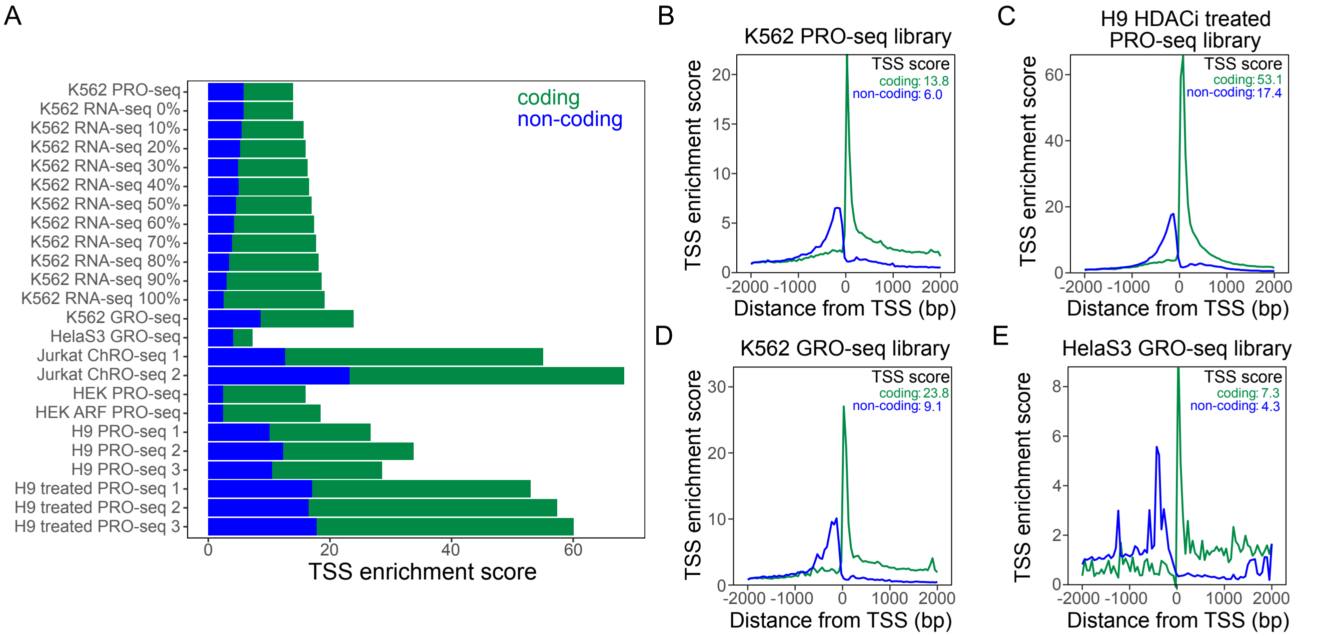

Fig. S5: ***TSS enrichment****. A) TSS enrichment scores for test set samples. B) Representative high-quality PRO-seq TSS enrichment plot. C) TSS enrichment plot in romidepsin treated PRO-seq library. D) Representative high quality GRO-seq TSS enrichment plot E) Representative example of lower quality GRO-seq TSS enrichment plot.*

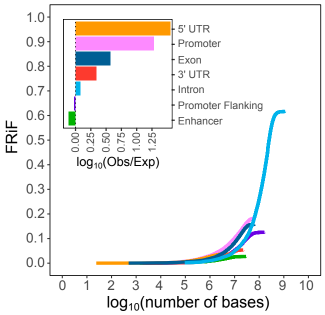

Fig. S6: ***Fraction of Reads in Features in ChRO-seq****. Cumulative FRiF and FRiF (inset) plots for example Jurkat ChRO-seq 1 library test sample shows increased enrichment of promoter sequences.*

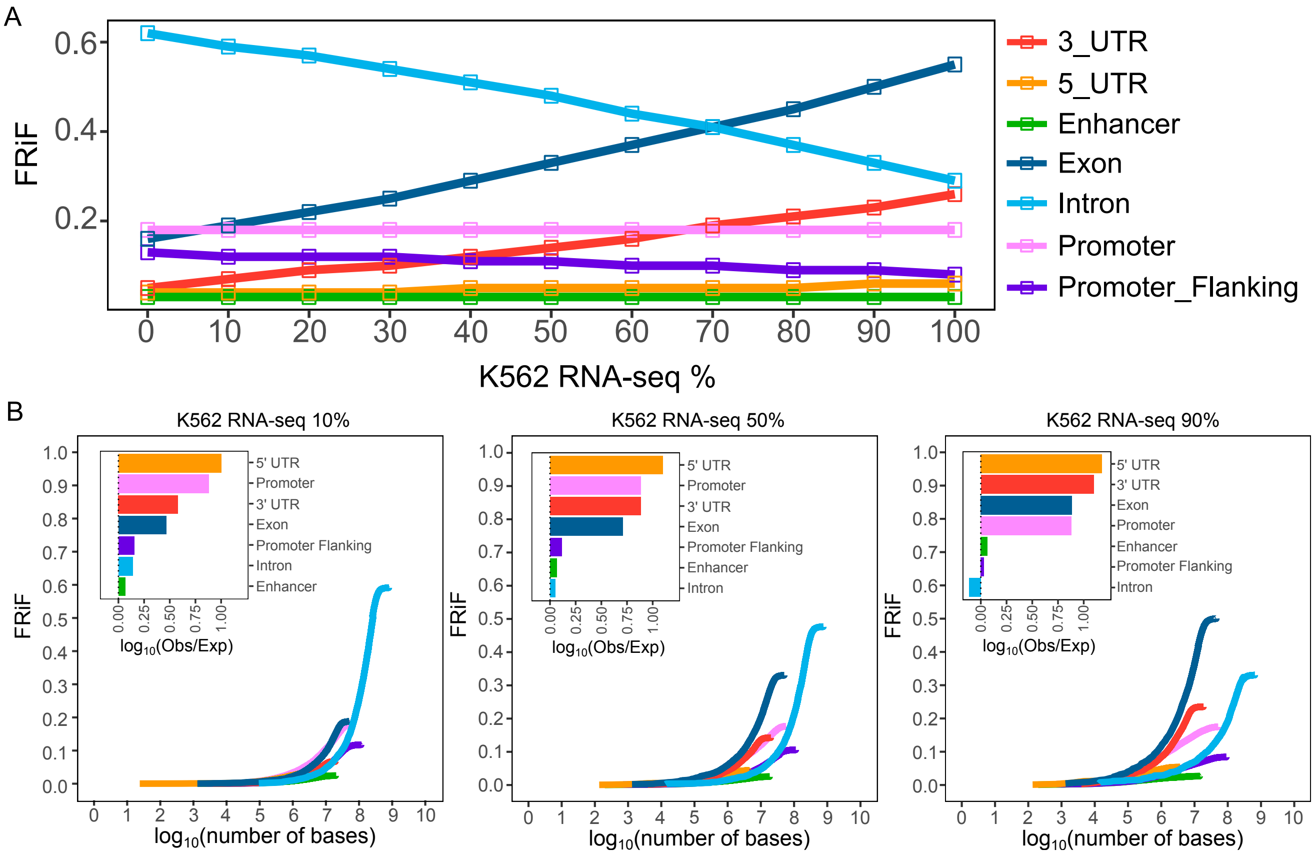

Fig. S7: ***RNA-seq spike-in shows how FRiF changes with mRNA contamination***. *A) Increasing percentages of RNA-seq spike-in lead to changes in the fraction of reads in features (FRiF). B) Cumulative FRiF plots at 10%, 50%, and 90% RNA-seq spike-in. Plot insets represent the expected versus observed fraction of reads in genomic features. Each spike-in library contains 70 million total reads.*

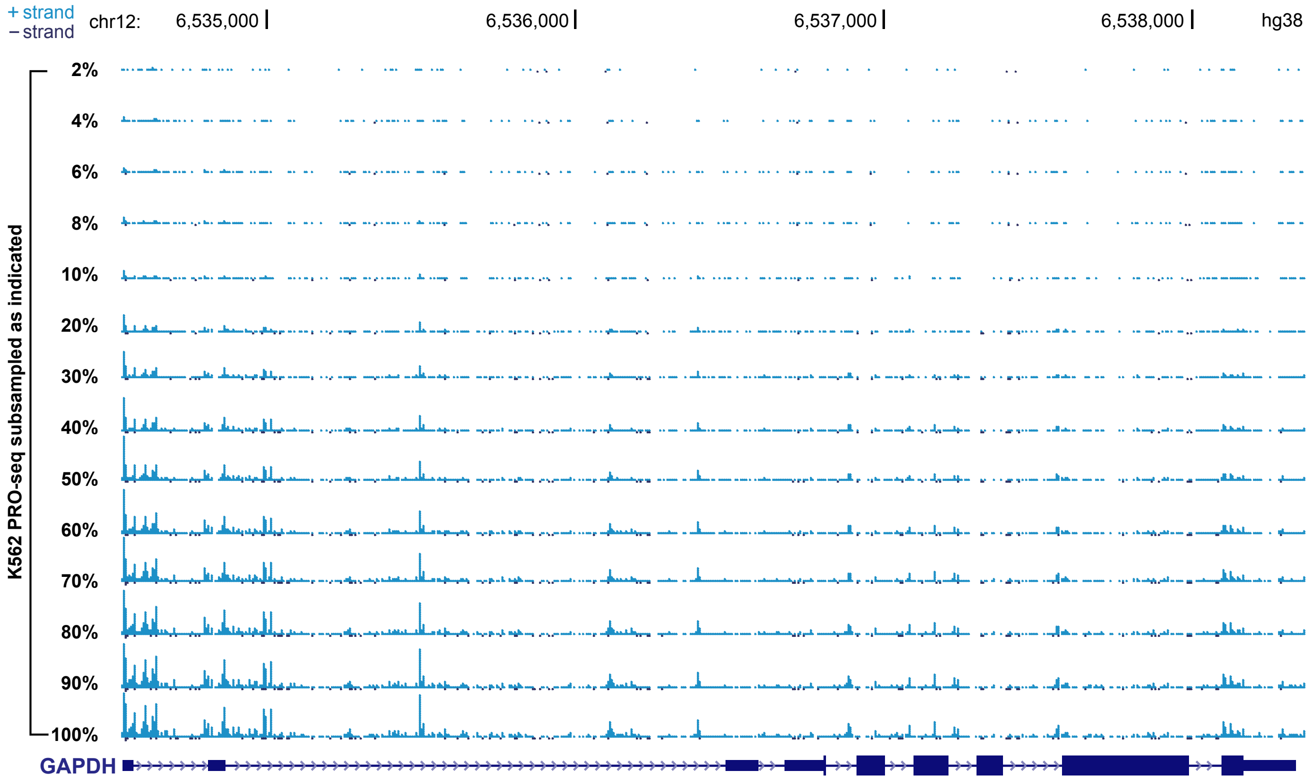

Fig. S8: ***K562 PRO-seq signal tracks show increasing coverage with depth***. *Incrementally subsampled K562 PRO-seq library signal tracks display reduced relative coverage at a representative locus (GAPDH). Fixed scale for each track is 100 to -10.*

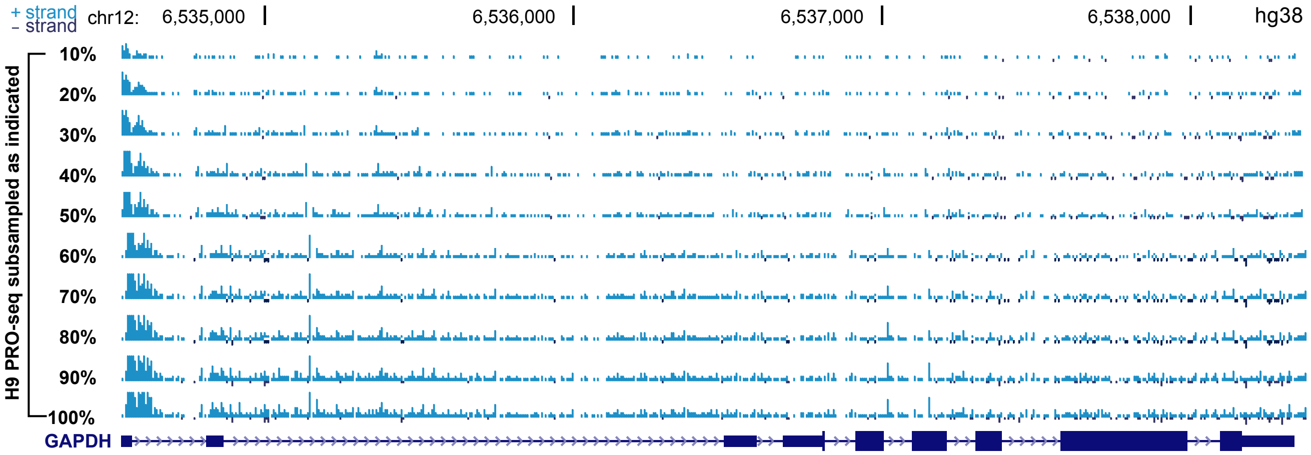

Fig. S9: ***H9 PRO-seq 2 signal tracks show increasing coverage with depth***. *Incrementally subsampled H9 PRO-seq 2 library signal tracks display reduced relative coverage at a representative locus (GAPDH). Fixed scale for each track is 10 to -5.*

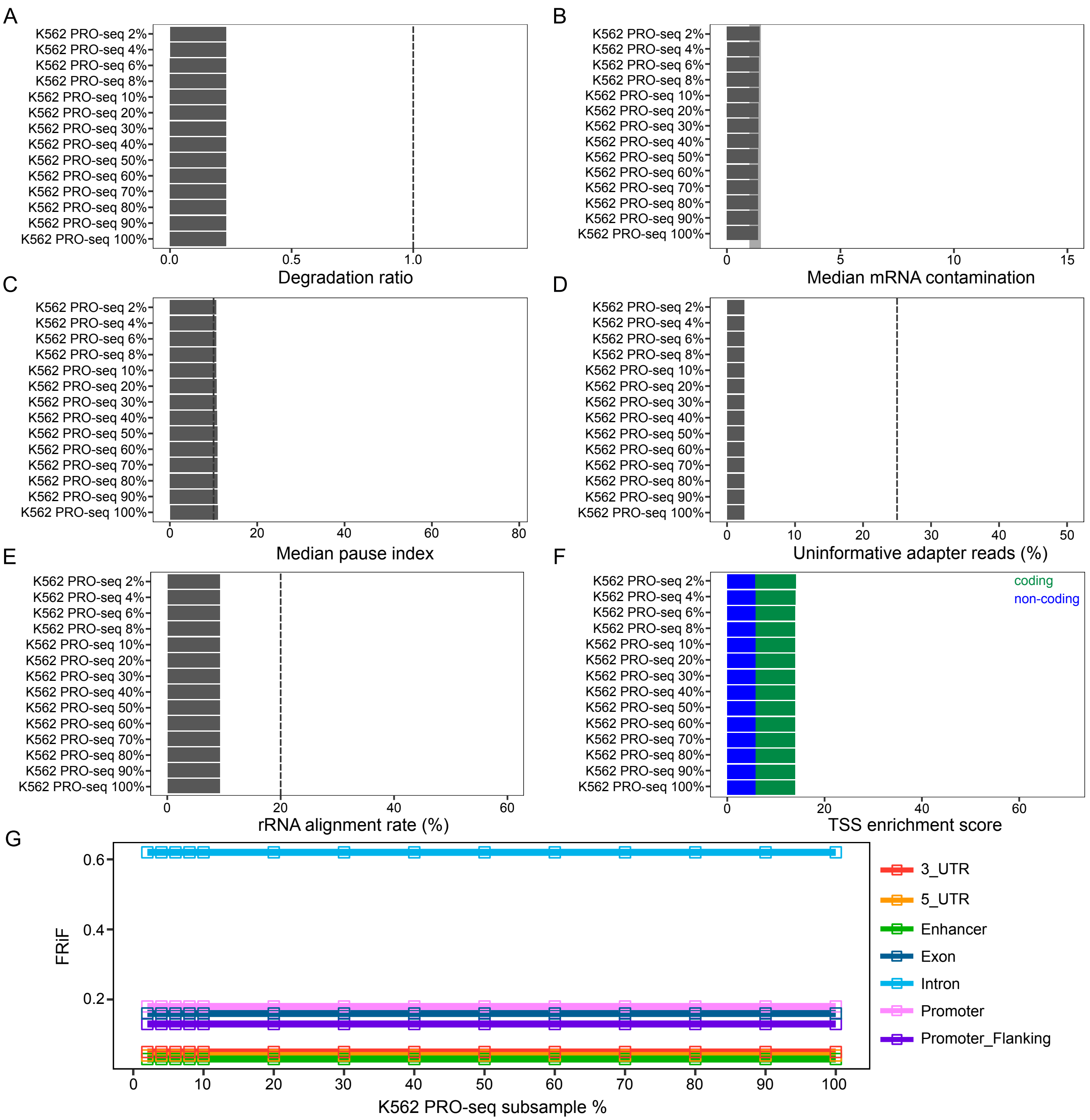

Fig. S10: ***QC metrics are not affected by sequencing depth in subsampled K562 PRO-seq****. Using subsampled K562 PRO-seq data, we show how various metrics behave across a spectrum of sequencing depths: A) Degradation ratio, B) mRNA contamination, C) Pause index, D) the percentage of uninformative adapter reads, E) the rDNA alignment rate, F) and the TSS enrichment scores are unaffected by sequencing depth. G) The FRiF and cumulative FRiF is unaffected by sequencing depth. The complete K562 PRO-seq library (100%) contains approximately 497 million reads.*

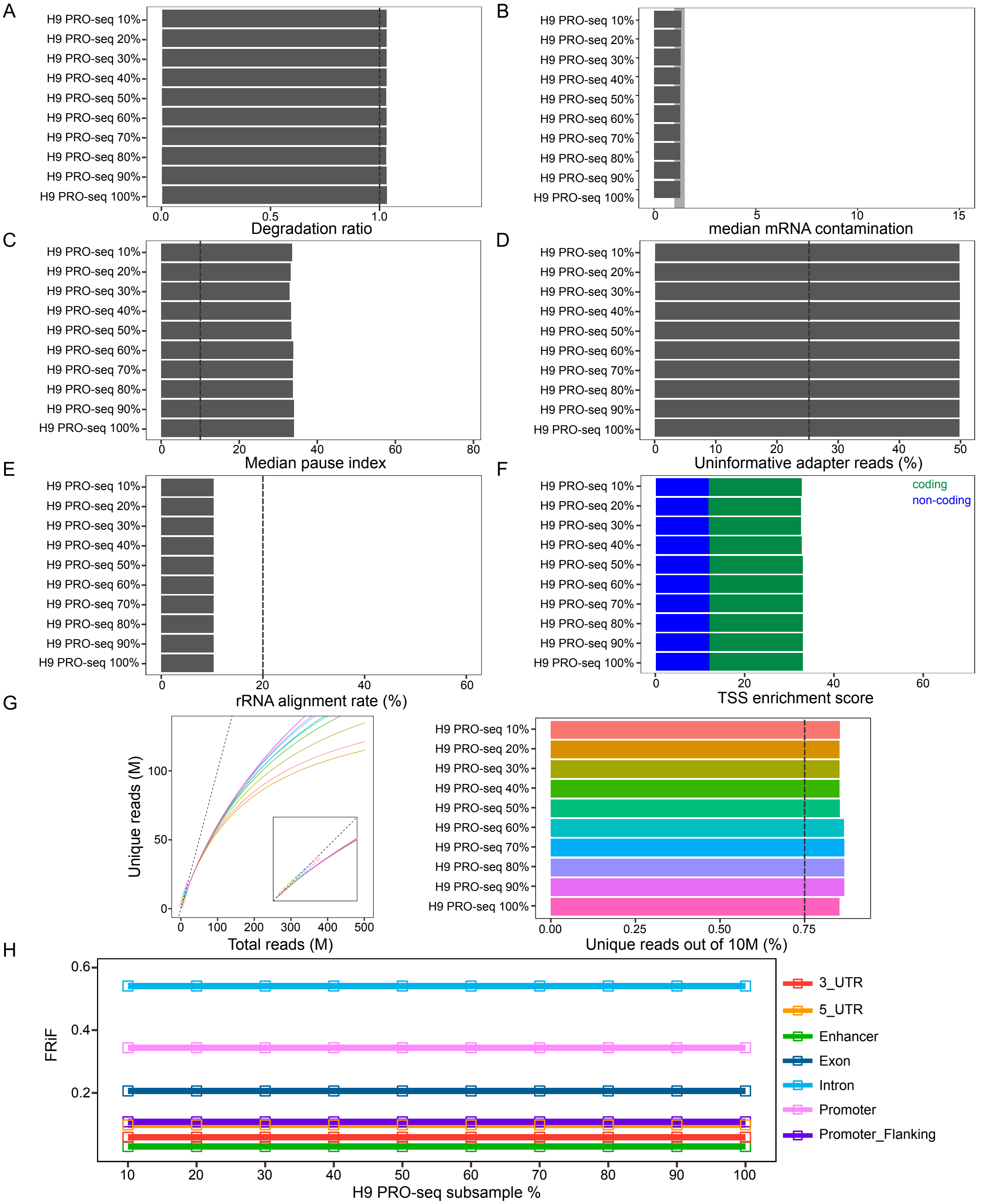

*Fig. S11: **QC metrics are not affected by sequencing depth in subsampled H9 PRO-seq**. Using subsampled H9 PRO-seq data, we show how various metrics behave across a spectrum of sequencing depths: A) Degradation ratio, B) mRNA contamination, C) Pause index, D) the percentage of uninformative adapter reads, E) the rDNA alignment rate, F) and the TSS enrichment scores are unaffected by sequencing depth. G) Library complexity traces plot the read count versus externally calculated deduplicated read counts. Deduplication is a prerequisite, so these plots may only be produced for samples with UMIs. Inset zooms to region from 0 to double the maximum number of unique reads. The position of curves in the left panel at a sequencing depth of 10 million reads (dashed line represents minimum recommended percentage of unique reads). H) The FRiF and cumulative FRiF is unaffected by sequencing depth. The complete H9 PRO-seq library (100%) contains approximately 116 million reads.*

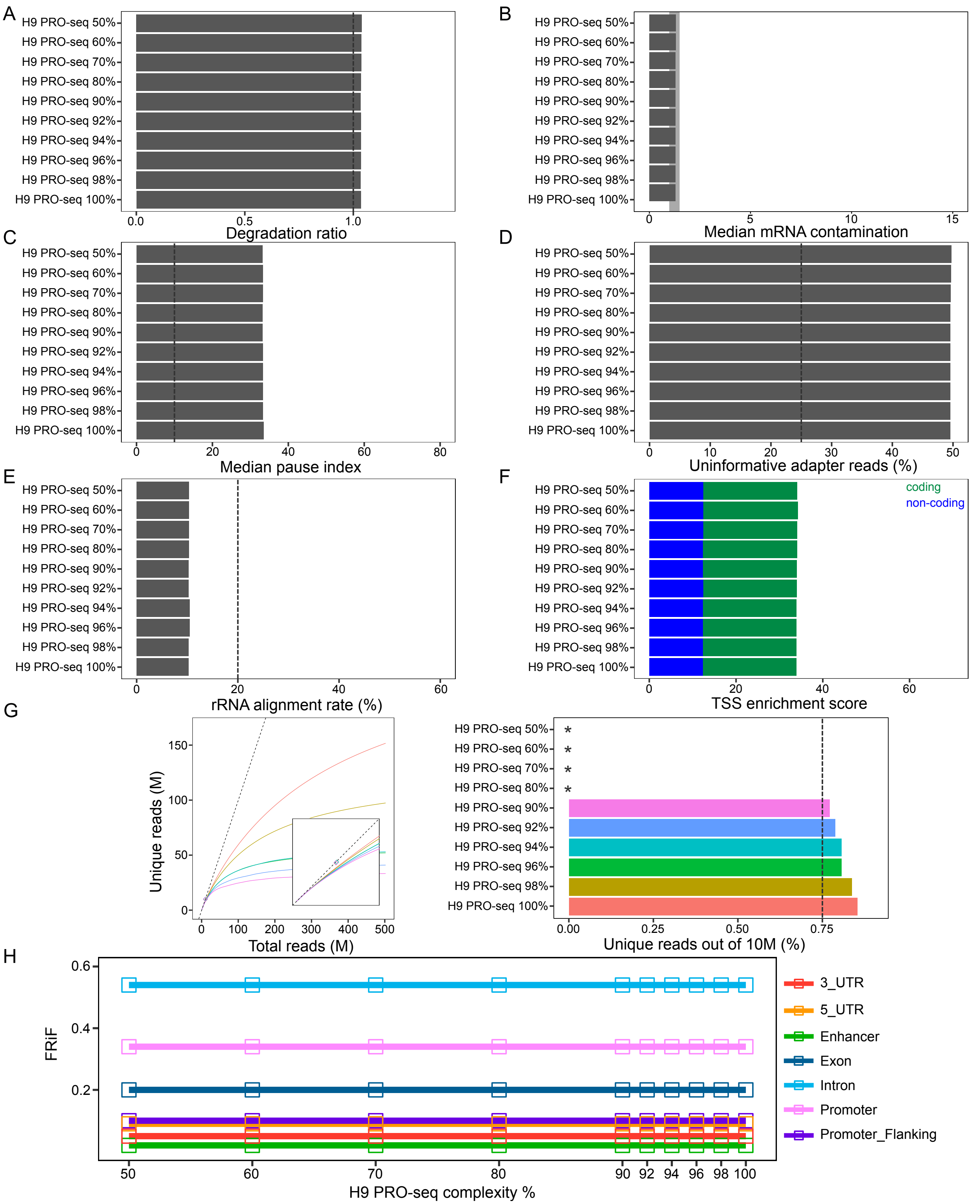

Fig. S12: ***QC metrics are not affected by low library complexity.*** *Using a synthetic set of libraries, we show how various metrics behave across a spectrum of complexity: A) Degradation ratio, B) mRNA contamination, C) Pause index, D) the percentage of uninformative adapter reads, E) the rDNA alignment rate, F) and the TSS enrichment scores are unaffected by low complexity. G) Library complexity traces plot the read count versus externally calculated deduplicated read counts. Deduplication is a prerequisite, so these plots may only be produced for samples with UMIs. Inset zooms to region from 0 to double the maximum number of unique reads. The right panel represents the position of curves in the left panel at a sequencing depth of 10 million reads (dashed line represents minimum recommended percentage of unique reads). *Libraries with less than 90% uniqueness could not be extrapolated due to saturation. H) The FRiF and cumulative FRiF is unaffected by low complexity. Each library contains 30 million total reads.*

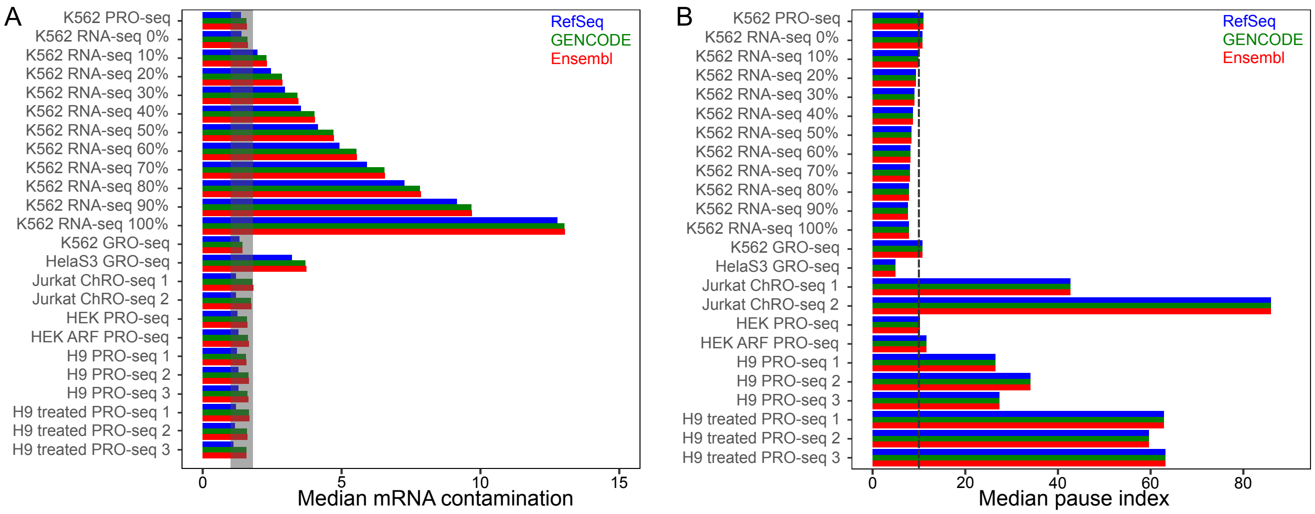

Fig. S13: ***Alternate annotation sources do not affect mRNA contamination and pause index****. A) The mRNA contamination metric and B) the pause index metric are robust across annotations.*
