# Supplementary material for: PEPPRO: quality control and processing of nascent RNA profiling data
Source: Genome Biol. 2021 May 15;22:155. doi: 10.1186/s13059-021-02349-4 (PMC8126160; doi:10.1186/s13059-021-02349-4)
Supplement: Supplementary file 2 — Additional file 2 Review history. [file 13059_2021_2349_MOESM2_ESM.docx]

**1^st^ round**

**Reviewer 1**

Ultimately this paper presents PEPPRO, which they claim has utility as two separate things: (1) a scalable workflow for nascent transcription and (2) novel analytical methods. Their pipeline is built over a collection of inhouse tools/approaches. It isn't clear what value it brings beyond similar pipelines built using existing pipeline frameworks. In contrast, their metrics have some potential to be of broad interest to the nascent transcription community, but they are not presented here in sufficient detail to understand their robustness to avarious modes of nascent transcription failure (poor complexity, low sequencing depth).

On the pipeline:

PEPPRO is a python library built over other libraries from the same group (pypiper, bulker, looper, refgenie).

The software itself, PEPPRO, is predominantly a pipeline tool. It's highly sequential, taking a parallelism by sample approach, with each sample getting its own pipeline that managed by a job manager (looper, another of their packages). The PEPPRO pipeline itself is a bunch of helper code followed by a large main function that builds shell commands as strings before submitting them to the job manager and waiting.

Installation is somewhat complicated by the multiple dependencies, many of them other software pacakges by the same group. Running PEPPRO in a container was somewhat simpler, but still required installation of bulker (one of their packages). Installing without a container will undoubtedly be restricted to large scale compute environments because of the large number of dependencies. Consequently, the target audience is not general biologists but rather bioinformatics groups looking for a pipeline / analysis approach for nascent transcription.

(*) They advocate for their pipeline to be restartable, fault tolerant, modular, and scalable (largely provided by pypiper). But unclear the advantage of this as compared to other bioinformatics pipeline frameworks (there are many including Nextflow (doi: 10.1038/nbt.3820) and SnakeMake; many others, reviewed at: https://doi.org/10.1093/bib/bbw020).

(*) The basics of the pipeline (Figure 1) are sound, though they discuss usage of an outdated mapping strategy (bowtie2 over HISAT2). While I know they say the pipeline is modular, defaults are still important as they are implicitly recommendations.

On the metrics:

(*) Reference genome information is provided by refgenie but not described for the examples they show (beyond that it's hg38). For example, exon-intron ratios must inevitably depend on the annotation set. While for human it's likely there isn't a huge difference in RefSeq vs ENCODE (for example), it is possible that their recommendations (1.1-1.4 ratio) may be sensitive to the annotation set. So showing the metrics are somewhat robust to annotation set is important.

(*) Their degradation ratios is a hack -- though still potentially informative. Both it and the adapter-adpater ligation metric depends on the size selection step of the protocol. This is discussed in the methods, but should be more up front in the main text. From a data reuse perspective, this is also concerning since sometimes publishers fail to mention these key details. So I inherently wonder if they can detect (directly from the data) when these metrics "fail".

(*) They mention complexity (Figure 4) but only calculate this for samples with UMIs. In my experience, most existing nascent data lacks UMIs (many recent publications do have it). In their colleciton only the K562 experiments (GRO and PRO) lack UMI. But invitably some of their metrics (and recommendations) may be sensitive to having highly complex libraries. It would be more informative to include poor complexity libraries (which are not uncommon in nascent -- it's a protocol that is prone to loss issues) to understand how their other metrics are influenced by this variable.

(*) Likewise the other "controllable" variable is depth. They mention it with respect to how size selection skews their adapter metric and degradation hack, but they don't otherwise discuss. Yet a rarefraction (similar to their variable injection of RNA-seq but now take a high quality dataset and subsample to various depths) would be simple to include.

(*) Every metric could be used independently of their pipeline if sufficient descriptions are provided. They mostly do a good job of this, though they do not explicitly describe the TSS enrichment score (presumably its just a ratio, but should be given).

(*) Their bigWig files report 3' end of reads only -- yet some downstream analysis software prefers full read. So this should be available as an option.

(*) They mention that you can optionally use seqOutBias -- but is this recommended? What is the ultimate effect of this normalization on nascent? In a paper where they are providing recommendations -- even a casual mention can be construted as a recommendation.

(*) The following statement is unclear "PEPPRO defines the density within the pause region as the single, most dense window +20-120 bp take from all annotated TSS per gene." Do they mean the window specified and that its *usually* the most dense?

(*) Figure issues:

-- Figure 2A labels samples 6 and 7 with one HEK group, but using numbers in parenthesis could be mistaken for citations, so should just describe these on lines as was done for samples 1-5.

-- Figure 2B should describe the colors -- presumably light blue is positive strand and dark blue is negative strand.

-- Figure 3 -- coloration in C-F is not described.

-- In all metric plots contrasting samples, the desired cutoffs should be noted with dashed lines or some other visual cue. For example, place on Fig 3B -- dashed line at 1; Figure 5B colored zone from 1.1-1.4; Figs S3, S4A

-- Fraction of reads over genomic features should be scaled consistently (x and y axis) throughout (Figure 7, S6).

-- Figure 8A is uninterpretable as its not know what individual dots correspond to -- annotated genes? eRNAs? isoforms? all of the above?

-- Figure S1 appears to be a lowly transcribed gene in K562 (top line). A second example with a more highly transcribed gene would greatly improve this figure.

(*) There are numerous capitalization issues in the citations (possible LaTex-isms): RNA, RNA pol II, DNA, etc.

(*) The supplemental text showing R code for constructing the DEseq data table is useful, but could be improved by documentation/descriptions for each line.

**Reviewer 2**

The paper by Simith et al addresses a topic to whom could be interested in GROseq data analysis. I think PEPPRO could be a useful tool to analyze many kinds of nascent RNA-seq data but the audience for such tool is, perhaps, not so wide to merit a Genome Biology publication. In any case I consider that the paper can be improved in some aspects that I detailed below:

1) Page 1 line 46. nascent RNA profiling measures orientation of transcripts but RNAseq can do it as well. Therefore, this cannot be considered as an advantage.

2) Why the PEPPRO is not used with other variant techniques? There are many other variants, apart from PROseq, GROseq and ChROseq, that could be tested (see Wissink et al., Nat Rev. Genet. https://doi.org/10.1038/s41576-019-0159-6 and Jordán-Pla, et al. ISBN 978-1-4939-3079-1_8 as recent reviews).

3) The use of Pausing as a test for Run-on efficiency is not clear to me. Not all genes have paused RNA polymerases. In fact they are, probably less than the ones not having it. Therefore, it only serves for those genes. Moreover, I cannot see what is the Run-on efficiency parameter for. Does it help in the data analysis?

4) Which programs were used for trimming and reverse complement? Cite them.

5) Why to use the experiment of HDAC inhibition as a control for pausing index? Is there any previous indication of that? Please clarify.

6) The plots in Figures 2B, 8c and S1 seem not to be normalized. It would be much better for comparison between samples to use RPKM instead of count number.

7) Fig. 3D: Substitute "size of insertion" by "insert size"

8) Fig. 3A: Substitute "20 bp protected RNA" by "20 b protected RNA". In The Graph on the right 20 bp can refer to dsDNA insert size but on the RNA pol scheme it is obviously wrong.

9) Page 3 line 53-54 Right column and many other locations: How do you define "high quality libraries". This statement should be done according to previously defined rules.

10) Fig. 5 legend: Part F is erroneously written as E.

11) Fig. 5E: The title K562 RNAseq 30% library is perhaps wrong according to the legend: K562 PROseq 30% library seems to apply.

12) Page 5 lines 43-44: Why do you state that over representation of rDNA reads means mature RNA contamination? Is is true that rRNA is much more abundant than mRNA but it is also true that rRNA transcription by RNA pol I is also higher than RNA pol II transcription. Therefore, this seems not to be a good estimation of mature RNA contamination.

13) Page 5 line 52-53: why the ratio between intron/exon reads should be 1 in a non-contaminated library? The proportion of intron/exon lengths is variable depending on genes so the proportion for a given gene when only measuring nascent RNA will vary. I do not know how is the average of the genome for intro/exon total lengths but is difficult to think that it is precisely 1.

14) The "in silico" spike-in is not the optimum test. It would be much better to have done a "real" spike in wet experiment.

15) Page 7. Pipeline implementation. Please explain which were the "sensible defaults" used and describe more in depth the software used.

16) The web server provides a "summary table" with 80 columns that is not very user friendly

17) -Broken links: in the output directory of the web, for example in the reports folder. In the Extended tutorial, point 5, browse output page.

18) What is the output of running the pipeline directly (without Looper). Is it an html file too?

19) Please consider extending the methods section to detail the tools used for pre-processing, alignment and coverage track generation, as well as a brief recap of the default parameters used (e.g. how are reads filtered: quality, size, both?; what is the multi mapping criteria during alignment?, etc).

20)Consider making the count table as a discrete plain text file in the output folder.

**Author response**

**Reviewer 1**

Ultimately this paper presents PEPPRO, which they claim has utility as two separate things: (1) a scalable workflow for nascent transcription and (2) novel analytical methods. Their pipeline is built over a collection of inhouse tools/approaches. It isn’t clear what value it brings beyond similar pipelines built using existing pipeline frameworks.

There are many general-purpose RNA-seq pipelines, of course, using many different pipeline frameworks – but unfortunately, the plentiful and powerful RNA-seq pipelines cannot be applied to nascent RNA-seq data effectively. **We are not aware of a single published pipeline for nascent RNA-seq data.** To our knowledge, PEPPRO is the first PRO-seq pipeline to be built to the standards of community reproducibility and publication. Currently, nascent RNA-seq papers typically run in-house scripts to process data, which are generally not well explained, difficult to reproduce, and poorly documented. In our opinion, the community benefits from dedicated effort to produce a robust, standardized pipeline. It has given us opportunity to develop dedicated metrics and dedicated tooling that can establish a starting point for more detailed analysis of nascent RNA data.

In contrast, their metrics have some potential to be of broad interest to the nascent transcription community, but they are not presented here in sufficient detail to understand their robustness to a various modes of nascent transcription failure (poor complexity, low sequencing depth).

We appreciate the acknowledgment of the utility of our metrics. We have now increased the detail in our descriptions of our metrics, and expanded the set of test cases to show how they behave in a wider range of experimental data (complexity, sequencing depth). We outline these improvements in more detail in specific responses below.

On the pipeline:

PEPPRO is a python library built over other libraries from the same group (pypiper, bulker, looper, refgenie). The software itself, PEPPRO, is predominantly a pipeline tool. It’s highly sequential, taking a parallelism by sample approach, with each sample getting its own pipeline that managed by a job manager (looper, another of their packages). The PEPPRO pipeline itself is a bunch of helper code followed by a large main function that builds shell commands as strings before submitting them to the job manager and waiting. Installation is somewhat complicated by the multiple dependencies, many of them other software packages by the same group. Running PEPPRO in a container was somewhat simpler, but still required installation of bulker (one of their packages). Installing without a container will undoubtedly be restricted to large scale compute environments because of the large number of dependencies. Consequently, the target audience is not general biologists but rather bioinformatics groups looking for a pipeline / analysis approach for nascent transcription.

Thanks for raising this issue, and we appreciate your trying out both the containerized and native installs of PEPPRO. Pipeline dependencies are a tricky issue, of course, that the whole community is struggling with right now. Containers are a promising approach, and while they’ve solved many problems, they bring their own set of challenges. We have taken great effort to make PEPPRO usable either with containers or natively. In our opinion, the flexibility offered by PEPPRO is an advantage. The container approach is simpler for those familiar with containers, and it is what we use for our analysis, but they have a learning curve that dissuades many.

As for the number of dependencies, like many sequencing processing pipelines, PEPPRO depends on lots of existing tools. These reflect the complexity of the analysis, plus the complexity of the quality control metrics we use. Any pipeline that performed the analysis done by PEPPRO will have a similar dependency list. But while we advocate using the container approach, installing without containers is less difficult than it may appear at first. In our experience, people are willing to invest a bit of time in setup if they can get the highest quality processing results, like those provided by PEPPRO. For labs that regularly process sequencing data, many of these tools are routine and will be already available on local or HPC systems. Furthermore, since PEPPRO includes options for many of the analyses, you only have to install the ones you select. So, the dependency list is not as onerous as it may first seem.

Nevertheless, we acknowledge the criticism, and have thoughtfully considered how to minimize, present, and document dependencies. We reconsidered the way we distributed tools and presented optional tools. To address this, we have revamped the dependency structure and deployed some of our internal software onto accepted channels (CRAN, PyPI). This effort has simplified the native installation, making PEPPRO approachable for individual labs. We have one remaining package (GenomicDistributions) that is awaiting acceptance into Bioconductor, which will simplify installation steps even further. We appreciate this critique, as it inspired us to rethink the installation procedure. We believe we have made the pipeline more approachable and will continue to work to simplify the installation steps.

(*) They advocate for their pipeline to be restartable, fault tolerant, modular, and scalable (largely provided by pypiper). But unclear the advantage of this as compared to other bioinformatics pipeline frameworks (there are many including Nextflow (doi: 10.1038/nbt.3820) and SnakeMake; many others, reviewed at: https://doi.org/10.1093/bib/bbw020).

There are many ways to make useful pipelines, including Nextflow and Snakemake as you mention, which are great tools that produce robust pipelines. In fact, we are working with the developers of both Nextflow and Snakemake on other, unrelated projects related to distributing standardized reference assembly files and metadata descriptions. In any case, our intent here was not to publish a pipeline framework, but a particular pipeline, and we made no claims about relative merits of pipeline frameworks – we believe there are many valid choices that would satisfy the requirements of being restartable, fault tolerant, modular, and scalable. We merely wanted to emphasize that we have done that. We have simplified parts of the introduction to make sure they are not misconstrued as a claim about merits of a particular pipeline framework, and we think PEPPRO should be published on the merits of the pipeline itself, not on the tools used to build it.

(*) The basics of the pipeline (Figure 1) are sound, though they discuss usage of an outdated mapping strategy (bowtie2 over HISAT2). While I know they say the pipeline is modular, defaults are still important as they are implicitly recommendations.

Thank you, we appreciate the recognition that the pipeline steps are sound. For alignment, since we are measuring nascent transcription, we opted to implement an alignment strategy that was conventional for genome alignments as opposed to spliced transcriptome alignments. Although we appreciate that hisat2 can align directly to a genome, in our experience, and in consultation with the authors of the tool, bowtie2 is still preferred for conventional alignment (eg https://doi.org/10.1101/657874). We have also had some technical issues with the use of hisat2, specifically the script “hisat2 read statistics.py”, with regards to the latest python3 installation (specifically line 182 syntax is no longer valid with non-reverse compatible python3). Thus, while we acknowledge the clear value of hisat2, bowtie2 remains a perfectly valid mapping strategy for genomic reads.

On the metrics:

(*) Reference genome information is provided by refgenie but not described for the examples they show (beyond that it’s hg38). For example, exon-intron ratios must inevitably depend on the annotation set. While for human it’s likely there isn’t a huge difference in RefSeq vs ENCODE (for example), it is possible that their recommendations (1.1-1.4 ratio) may be sensitive to the annotation set. So showing the metrics are somewhat robust to annotation set is important.

Thank you for the suggestion. We have now added a description of the annotation we used, which is dependent on the metric. For the exon:intron density ratios we use the UCSC RefSeq gene annotation as the source. For the pause index we use Ensembl annotation files as the source. To illustrate differences due to annotation source, we now include comparisons for these metrics using the RefSeq, Ensembl, and GENCODE annotations. We have added a note to the effect that our recommendations depend on the reference data used. We also wanted to point out that the exon:intron ratio is not read counts in exons vs. introns, but rather, the **density** of reads within exons and introns. In other words, our metric corrects for annotation size. Since nascent RNA density should be relatively constant throughout the gene body, this metric is not likely to change much between annotation sets. To confirm this, we tested the metric with different annotation sets and found that this metric is robust to different annotation sets. We have added a new supplemental figure and discussion to demonstrate this (Fig. S12).

(*) Their degradation ratios is a hack – though still potentially informative. Both it and the adapter-adpater ligation metric depends on the size selection step of the protocol. This is discussed in the methods, but should be more up front in the main text. From a data reuse perspective, this is also concerning since sometimes publishers fail to mention these key details. So I inherently wonder if they can detect (directly from the data) when these metrics “fail”.

We are not sure exactly what you mean by “hack”. It is very true that publishers often fail to mention key details, such as size selection – in fact, this really underscores the value of the metric, because it can be used to hint at these kinds of steps, even if papers don’t specify exactly what they did. Thus, we don’t see this as a case where the metric “fails” – on the contrary, we see this as one of the scenarios the metric can identify! As you suggest, in a way, our metrics provide us a way to learn about the actual protocol used from the data itself, so that we don’t have to rely on published protocol descriptions, which may be incomplete or incorrect. While many PEPPRO users are likely to run the pipeline on their own data, for which they presumably have pretty reliable protocol information, we also envision PEPPRO being run on publicly available data, where that kind of information is less polished. In this case, we can simply run the metrics as normal, and they provide a lot of information about what likely steps, such as size selection, went into the protocol.

A bit more detail about size selection in particular: we do not recommend size selection on a nascent RNA-seq library beyond excluding the adapter/adapter ligation product. Many nascent RNA species are in the range of 20 - 30 bases, so there is biological reason for no size selection. So, whether a library is inherently biased and “fails” can best be determined directly from the data.

In terms of the adapter/adapter ligation product, the metric does not inform on the biological interpretation of the data; it merely allows the user to determine the rate at which higher sequencing depth results in informative reads vs. uninformative adapter reads. As requested, we have made more prominent the discussion of the size selection step.

(*) They mention complexity (Figure 4) but only calculate this for samples with UMIs. In my experience, most existing nascent data lacks UMIs (many recent publications do have it). In their collection only the K562 experiments (GRO and PRO) lack UMI. But inevitably some of their metrics (and recommendations) may be sensitive to having highly complex libraries. It would be more informative to include poor complexity libraries (which are not uncommon in nascent – it’s a protocol that is prone to loss issues) to understand how their other metrics are influenced by this variable.

Thank you for the suggestion. To address this, we have produced and analyzed several synthetic low-complexity libraries in the examples. Furthermore, your suggestion below of a sampled library also addresses this point, and we have added this as well. Figure S11 now shows how a synthetic low complexity library behaves across the metrics. Our conclusion is that our library complexity metrics capture the simulated library complexity differences, and that all other metrics are robust to the library complexity differences. Together, these updates make it much easier to see how library complexity influences all of our metrics. This suggestion has added utility to the paper.

Regarding the calculation of library complexity from UMIs – you are correct that much of the early nascent RNA data lacked UMIs. Unfortunately, it is not possible to calculate complexity without UMIs due to the biology of nascent RNA-seq. For conventional RNA-seq, shearing is random, so paired end reads that have the same start and end coordinate would be unlikely to be real and are typically assumed to be PCR duplicates. However, in nascent RNA-seq, RNA polymerase II promoter proximal pausing causes many independent reads to have the same 5’ end (the transcription start site) and the same 3’ end (the paused region). As a result, nascent RNA reads cannot be effectively deduplicated using the standard approach applied to traditional RNA-seq. This is why we only calculate complexity for samples with UMIs. Of course, it would be nice to do otherwise, but this is a fundamental limitation of the molecular biology. As you stated, many recent nascent RNA publications are now including UMIs for exactly this reason. We have addressed this confusion by clarifying further in the ‘Library complexity’ section.

(*) Likewise the other “controllable” variable is depth. They mention it with respect to how size selection skews their adapter metric and degradation hack, but they don’t otherwise discuss. Yet a rarefraction (similar to their variable injection of RNA-seq but now take a high quality dataset and subsample to various depths) would be simple to include.

Thank you for this suggestion. To address this, we have now added new studies where we subsampled high complexity libraries to show how depth influences the metrics. To evaluate the robustness of our metrics across sequencing depth, we ran PEPPRO on subsampled single-end and paired-end with UMI PRO-seq libraries. This analysis revealed that the pause index calculation indeed varied with depth as you predicted (Fig. R1C). To address this, we found that lowly expressed genes represent a significant portion of genes with a low pause index. At lower sequencing depth, these lowly expressed genes experience greater dropout and fluctuation in pause index calculation, skewing the metric upwards at low depth. To address this, we now restrict the pause index calculation to the upper 50th percentile of genes by expression, which eliminates the variability due to depth (Fig. R2). We also tested all the other metrics in the process. We observed that every other metric remained constant across sequencing depth from fewer than 10M reads to well over 100M (Fig. S9, Fig. R2).

In conclusion, in response to these two critical points, we added 2 new experiments: a simulated complexity series, and a simulated depth series, with new supplemental figures that nicely demonstrate that all of our metrics are robust to these two “controllable” variables. **We greatly appreciate this suggestion and this new analysis has added new support to the robustness of these metrics.**

(*) Every metric could be used independently of their pipeline if sufficient descriptions are provided. They mostly do a good job of this, though they do not explicitly describe the TSS enrichment score (presumably its just a ratio, but should be given).

Thanks. Yes, you are correct, these metrics are useful outside the pipeline. We consider the

refinement of these metrics to be a major contribution of our paper. We tried to make the

metrics clear by including a schematic diagram for each metric. We have also provided code

for calculating these metrics which can be used in other pipelines, if so desired. In the revision,

we have increased the description of all the metrics, and in particular have now fully described

the TSS enrichment score metric.

Fig. R1: **Original subsampled H9 PRO-seq**. A) Degradation ratio is unaffected by sequencing depth. B) mRNA contamination is unaffected by sequencing depth. C) Pause index undergoes small inflation at low sequencing depth. D) The percentage of uninformative adapter reads is unaffected by sequencing depth. E) The rDNA alignment rate is unaffected by sequencing depth. F) The TSS enrichment scores are unaffected by sequencing depth. G) Library complexity traces plot the read count versus externally calculated deduplicated read counts. Deduplication is a prerequisite, so these plots may only be produced for samples with UMIs. Inset zooms to region from 0 to double the maximum number of unique reads. The position of curves in the left panel at a sequencing depth of 10 million reads (dashed line represents minimum recommended percentage of unique reads). H) The FRiF and cumulative FRiF is unaffected by sequencing depth. The complete H9 PRO-seq library (100%) contains approximately 116 million reads.

Fig. R2: **Updated subsampled H9 PRO-seq**. A) Degradation ratio is unaffected by sequencing depth. B) mRNA contamination is unaffected by sequencing depth. C) Updated pause index is unaffected by sequencing depth. D) The percentage of uninformative adapter reads is unaffected by sequencing depth. E) The rDNA alignment rate is unaffected by sequencing depth. F) The TSS enrichment scores are unaffected by sequencing depth. G) Library complexity traces plot the read count versus externally calculated deduplicated read counts. Deduplication is a prerequisite, so these plots may only be produced for samples with UMIs. Inset zooms to region from 0 to double the maximum number of unique reads. The position of curves in the left panel at a sequencing depth of 10 million reads (dashed line represents minimum recommended percentage of unique reads). H) The FRiF and cumulative FRiF is unaffected by sequencing depth. The complete H9 PRO-seq library (100%) contains approximately 116 million reads.

(*) Their bigWig files report 3’ end of reads only – yet some downstream analysis software prefers full read. So this should be available as an option.

Because PRO-seq and similar methodologies sequence from the 3’ end, the bigWig files report reads **from** the 3’ end, but is not excluding any part of a read. If reporting from the 5’ end is required or preferred, the protocol for a sample may be set to ‘GRO-seq’, which does this automatically. Alternatively, the included bamSitesToWig tool may be run independently without the - -tail-edge option, and seqOutBias may also be run without the - -tail-edge option.

(*) They mention that you can optionally use seqOutBias – but is this recommended? What is the ultimate effect of this normalization on nascent? In a paper where they are providing recommendations – even a casual mention can be construed as a recommendation.

Thanks for this question, we have now added text in paper to clarify our recommendations. Correcting for enzymatic sequence bias is indeed important for nascent RNA-seq. As outlined in the seqOutBias paper, the bias associated with T4 RNA ligase can cause erroneous conclusions with regard to RNA polymerase transcription rate in the polypyrimidine tract and 3’ splice acceptor site. We clarify that we do recommend bias correction, especially when plotting composite profiles anchored on sequence features (e.g. transcription factor binding sites and splice acceptor sites).

() The following statement is unclear “PEPPRO defines the density within the pause region as the single, most dense window +20-120 bp take from all annotated TSS per gene.” Do they mean the window specified and that its usually* the most dense?

Starting with all annotated TSS’s for a gene, we define the pause window as the region +20- 120 bases from each identified TSS per gene. Then, we determine the coverage at every annotated pause window per gene, and identify the predominant, singular pause window as the pause window with the greatest coverage. That singular pause window is used to calculate the pause index for that gene. We understand the complexity of that statement and have added text in the body of paper regarding this.

(*) Figure issues:

Thank you for the detailed suggestions on the figures. We really appreciate these thoughtful details you’ve pointed out. We’ve now corrected all of these points and the resulting figures are much more interpretable now!

– Figure 2A labels samples 6 and 7 with one HEK group, but using numbers in parenthesis could be mistaken for citations, so should just describe these on lines as was done for samples 1-5.

Done!

– Figure 2B should describe the colors – presumably light blue is positive strand and dark blue is negative strand.

Labeled!

– Figure 3 – coloration in C-F is not described.

Added to legend!

– In all metric plots contrasting samples, the desired cutoffs should be noted with dashed lines or some other visual cue. For example, place on Fig 3B – dashed line at 1; Figure 5B colored zone from 1.1-1.4;

Figs S3, S4A

3B: done. 4C: added at 75% unique reads. 5B: added at 1 to 1.8. 6B: Added at 10. S2, S3, and S4 added.

– Fraction of reads over genomic features should be scaled consistently (x and y axis) throughout (Figure 7, S6).

We have now fixed the y axis scale at 1, as suggested. We have also fixed the x-axis scales. You are right, this makes these figures much easier to compare.

– Figure 8A is uninterpretable as its not know what individual dots correspond to – annotated genes? eRNAs? isoforms? all of the above?

The individual dots do correspond to annotated genes. This information has been added to the figure legend.

– Figure S1 appears to be a lowly transcribed gene in K562 (top line). A second example with a more highly transcribed gene would greatly improve this figure.

Our original example was, and continues to be, GAPDH, a known highly expressed gene. It probably appeared low because this was a subsampled experiment that we ran on only 10 million reads. We have now repeated this experiment with deeper sequencing (70M) and updated this figure. The tracks are also intentionally non-normalized to illustrate the increasing coverage at exons as the percentage of RNA-seq source reads increases.

(*) There are numerous capitalization issues in the citations (possible LaTex-isms): RNA, RNA pol II, DNA, etc.

These are indeed caused by LaTeX bibliography formatting, just due to the bibliography style we chose. We have now gone through our library to correct these kinds of capitalization errors.

(*) The supplemental text showing R code for constructing the DEseq data table is useful, but could be improved by documentation/descriptions for each line.

Added line-by-line documentation

**Reviewer 2**

The paper by Smith et al. addresses a topic to whom could be interested in GROseq data analysis. I think PEPPRO could be a useful tool to analyze many kinds of nascent RNA-seq data but the audience for such tool is, perhaps, not so wide to merit a Genome Biology publication. In any case I consider that the paper can be improved in some aspects that I detailed below:

Thank you for the comment and we are glad you think this topic is interesting to GROseq data analysis! PEPPRO can handle call kinds of nascent RNA data, which has been growing with recent improvements in the molecular biology. Most prominent among these is PRO-seq (Precision Run On), which improved upon the earlier GRO-seq protocols by providing base-pair resolution for where the RNA polymerase is located. The introduction of PRO-seq has enabled many new kinds of analysis and discoveries, such as identifying active regulatory elements, systematic characterization of transcription start sites, and genomic measurements of RNA Polymerase elongation rates. A more recent protocol advance is ChRO seq (Chromatin Run On), which has now expanded the nascent RNA protocols into additional sample types. The PRO-seq protocol continues to be improved (see A rapid, sensitive, scalable method for Precision Run-On sequencing (PRO-seq), https://doi.org/10.1101/2020.05.18.102277, Posted in May 2020, and Rapid and scalable profiling of nascent RNA with fastGRO, https://www.biorxiv.org/content/10.1101/2020.01.24.916015v1.full, posted in January). These recent improvements are leading to a new surge in interest in Run-on sequencing experiments and they provided the impetus for us to invest effort in building PEPPRO.

While the nascent RNA community may be smaller than the community for RNA-seq as a whole, these improvements are driving a lot of interest and so interest in PRO-seq and ChROseq are growing rapidly. However, there is currently a dearth of tools to process these new data

types. **PEPPRO is the first robust and polished pipeline for these data types. Given the growing interest in nascent RNA and the lack of existing analysis approaches, we feel that PEPPRO will be of widespread and growing interest in the next couple of years. Therefore, we feel that PEPPRO will have substantial impact on the community that warrants a publication in Genome Biology.**

Genome Biology recently published a review of ATAC-seq analysis and nascent RNA sequencing (according to a Web of Science search; term “nascent RNA sequencing” vs “ATAC-seq”) already has more total citations than ATAC-seq does (further breakdown: “ATAC-seq”:6,676 total; “PRO-seq”: 562 total, “GRO-seq”: 4,954 total,“ChRO-seq”: 19 total, and “Nascent RNA sequencing”: 81,129 total). Therefore, we argue it’s relevance is quite broad and appropriate to the journal.

**Specific comments**

1) Page 1 line 46. nascent RNA profiling measures orientation of transcripts but RNAseq can do it as well. Therefore, this cannot be considered as an advantage.

Yes, RNA-seq can measure transcript orientation; however, this depends on how the RNAseq library preparation step was performed. In our experience, many (even most) RNA-seq libraries are not processed in a way to preserve transcript orientation. In contrast, orientation is inherent in PRO-seq library construction. Therefore, all PRO-seq libraries measure transcript orientation by default, and we consider this an advantage. We have adjusted the text to clarify this claim.

2) Why the PEPPRO is not used with other variant techniques? There are many other variants, apart from PROseq, GROseq and ChROseq, that could be tested (see Wissink et al., Nat Rev. Genet. https://doi.org/10.1038/s41576-019-0159-6 and Jord´an-Pla, et al. ISBN 978-1-4939-3079-1 8 as recent reviews).

This is a fair point, but these are the most widely used methods and we know the molecular biology that goes into these methods much better than all the other techniques. We have tested PEPPRO on additional techniques, including NET-seq and TT-seq. Each of these techniques includes specific analysis variations that are not shared broadly among the other nascent sequencing methodologies. While the pipeline can successfully process these files as it stands, the specificities of the additional variants require manual tweaking. As these related technologies are yet to be as broadly adopted, we feel the additional intricacies do not merit inclusion by default at this time. However, we would be more than happy to work with users of these technologies to adapt PEPPRO for use should they grow in adoption.

3) The use of Pausing as a test for Run-on efficiency is not clear to me. Not all genes have paused RNA polymerases. In fact they are, probably less than the ones not having it. Therefore, it only serves for those genes. Moreover, I cannot see what is the Run-on efficiency parameter for. Does it help in the data analysis?

Most expressed genes are paused. The way we are using this metric, we consider the distribution across all genes. In short, polymerases in the gene body run on efficiently with poor run on conditions, but paused polymerases do not run on if conditions of high salt or high detergent are not met. This is outlined in detail in the original paper that characterized pausing (Rougvie and Lis, 1988) and genome-wide pausing (Core, et. al., 2012 Cell Reports). To emphasize this point, we included paired libraries: one that intentionally compromised polymerase run on conditions (no detergent) and a conventional GRO-seq library. The metric clearly distinguishes the run on lacking Sarkysol detergent.

4) Which programs were used for trimming and reverse complement? Cite them.

We have added citations to all software used either by default or optionally.

5) Why to use the experiment of HDAC inhibition as a control for pausing index? Is there any previous indication of that? Please clarify.

We apologize for the confusion here; the HDAC inhibition experiment was not included as a control for pausing index. It was included as a test to demonstrate differential expression analysis using PEPPRO. It also provided additional example libraries for the metrics in general. We have clarified the text to make clear the purpose of the HDAC inhibition experiment.

6) The plots in Figures 2B, 8c and S1 seem not to be normalized. It would be much better for comparison between samples to use RPKM instead of count number.

Yes, thank you for this observation as we agree. We have updated plots to include normalized signal tracks where relevant. In supplemental figure S1, we intentionally wish to illustrate the increasing depth at exons as RNA-seq read percentage grows.

7) Fig. 3D: Substitute “size of insertion” by “insert size”

Done!

8) Fig. 3A: Substitute “20 bp protected RNA” by “20 b protected RNA”. In The Graph on the right 20 bp can refer to dsDNA insert size but on the RNA pol scheme it is obviously wrong.

Agreed! Done.

9) Page 3 line 53-54 Right column and many other locations: How do you define “high quality libraries”.

This statement should be done according to previously defined rules.

Thanks for the suggestion. We adjusted the phrasing to make this more clear. We had intended to establish on case-by-case basis using the metrics to guide us.

10) Fig. 5 legend: Part F is erroneously written as E.

Fixed, thank you!

11) Fig. 5E: The title K562 RNAseq 30% library is perhaps wrong according to the legend: K562 PROseq 30% library seems to apply.

It actually is spiked with 30% RNA-seq. We are saying that it is composed of 30% RNA-seq reads, and the remainder is PRO-seq.

12) Page 5 lines 43-44: Why do you state that over representation of rDNA reads means mature RNA contamination? Is is true that rRNA is much more abundant than mRNA but it is also true that rRNA transcription by RNA pol I is also higher than RNA pol II transcription. Therefore, this seems not to be a good estimation of mature RNA contamination.

We find that no more than 20 percent of nascent RNA aligns to rDNA for high quality libraries. In contrast between 70 and 80 percent of mature RNA in a cell is rRNA. Therefore, while it is true that Pol I transcription is high, the relative ratio of rDNA aligned reads compared to the all other reads is an orthogonal metric to estimate mature RNA contamination. Here, we show that indeed, these two measures are significantly correlated (Fig. R3). Further, we have added text to clarify this point.

Fig. R3: **Abundance of rDNA to total reads is correlated with mature RNA contamination.** A) Correlation plot between the measure of mRNA contamination (median exon:intron density) and the ratio of rDNA aligned reads to total reads for all primary samples (*excludes RNA-seq spike-in experiment due to ribosomal depletion inherent in RNA-seq library preparation). B) Correlation plot between the measure of mRNA contamination (median exon:intron density) and the ratio of rDNA aligned reads to total reads for primary samples excluding the known outlier HelaS3 GRO-seq sample.

13) Page 5 line 52-53: why the ratio between intron/exon reads should be 1 in a non-contaminated library? The proportion of intron/exon lengths is variable depending on genes so the proportion for a given gene when only measuring nascent RNA will vary. I do not know how is the average of the genome for intron/exon total lengths but is difficult to think that it is precisely 1.

We apologize for this confusion, which was also raised by the other reviewer. The exon:intron ratio is not read counts in exons vs. introns, but rather, the **density** of reads within exons and introns. In other words, our metric corrects for annotation size. We also show how this metric may change slightly depending on the annotation source, but that the differences between samples remain constant.

14) The “in silico” spike-in is not the optimum test. It would be much better to have done a “real” spike in wet experiment.

We unfortunately were not easily set up to perform this as a wet experiment, however, the HelaS3 GRO-seq sample provides a “read-life” example of a contaminated experiment (indicating a roughly 30% mRNA contamination).

15) Page 7. Pipeline implementation. Please explain which were the “sensible defaults” used and describe more in depth the software used.

You are right about this and we have added additional information regarding default settings and all the required software, including necessary python packages.

16) The web server provides a “summary table” with 80 columns that is not very user friendly

The summary table reflects the sample annotation file, which is user dependent. The browsable HTML table also lets you choose to filter and only keep specific columns. However, we agree this is a lot of information to digest and as such, we have eliminated columns that could be found independently and do not add relevant information to this example.

17) -Broken links: in the output directory of the web, for example in the reports folder. In the Extended tutorial, point 5, browse output page.

Thank you for this catch, we have fixed the broken links.

18) What is the output of running the pipeline directly (without Looper). Is it an html file too?

The default output from the pipeline without looper does not include a HTML file. The HTML report is generated by looper, using the results of the pipeline as input. You could run the report generator from looper on just one sample, but its real value comes when you have a large project. With a single sample, it’s relatively easy to just browse the files in the folder. All sample QC plots and output files are generated by the pipeline – looper just aggregates the output into a single, easy to browse report.

19) Please consider extending the methods section to detail the tools used for pre-processing, alignment and coverage track generation, as well as a brief recap of the default parameters used (e.g. how are reads filtered: quality, size, both?; what is the multi mapping criteria during alignment?, etc).

Thank you for the suggestion. We now describe the bowtie2 settings in the serial alignment text section, and we indicate how samtools is used, including the specific tool parameter, to remove low-quality reads.

20) Consider making the count table as a discrete plain text file in the output folder.

The counts table is a plain text file. The project count table is saved as a CSV file in the summary/output folder and a link that file is accessible from the looper generated HTML report.

**2nd round**

**Reviewer 2**

The current revised version addresses many questions I raised previously but fails to correct some of them

1) Regarding my general question of the wide audience of a method for the bioinformatic analysis of PROseq and related methods I am not fully convinced by the authors arguments. It is true that PRO-seq is a common technique but they are only 3 publications using ChRO-seq (made by the inventors of it). On the contrary, NET-seq and TT-seq (not included in PEPPRO) are much more used. The use of ChRO-seq cannot be said to be "widespread".

2) The second advantage of PRO-seq over RNA-seq cited in the introduction is not true. RAN-seq can be made with orientation, as the authors acknowledge. Therefore, this is not an advantage. In fact, in can be said that RAN-seq has the advantage to allow the selection for orientation-specific or not whereas PRO-seq is inherent and obligatory to PRO-seq.

3) My previous question #6 related with the normalization in Figs. 2B, 8C and S1 is answered as "we have updated plots" but eh 2B & 8C plots are identical to the previous version. In the legend it is said they are normalized. Therefore, one can conclude that they were already normalized in the previous version. If that is true why the authors say the they updated the graphs? If not, why they show an identical plot to the previous unnormalized version?

4) My previous question #12 for the use of rDNA abundance as a control of mature RNA is not convincingly answered. The plot shown in Fig. R3 left shows a good correlation between rDNA level and mRNA contamination. However, the right graph shows that this good correlation strongly depends on the HeLaS3 point. The R goes bak to 0.48 and, importantly, the p-value is NOT significant (0.11)

**Author response**

**Reviewer 2**

The current revised version addresses many questions I raised previously but fails to correct some of them

1) Regarding my general question of the wide audience of a method for the bioinformatic analysis of PROseq and related methods I am not fully convinced by the authors arguments. It is true that PRO-seq is a common technique but they are only 3 publications using ChRO-seq (made by the inventors of it). On the contrary, NET-seq and TT-seq (not included in PEPPRO) are much more used. The use of ChRO-seq cannot be said to be “widespread”.

Indeed, ChRO-seq is a new technique that is very specialized and is not widely adopted yet. Our original sentence was unclear, as we intended to refer to PRO-seq with that comment in the introduction about these techniques being widespread. To address this, we have now couched that language in the introduction further. It now reads: “These advantages have led to growing adoption of global run-on (GRO-seq), precision run-on (PRO-seq), and, most recently, chromatin run-on (ChRO-seq) experiments”.

2) The second advantage of PRO-seq over RNA-seq cited in the introduction is not true. RNA-seq can be made with orientation, as the authors acknowledge. Therefore, this is not an advantage. In fact, in can be said that RNA-seq has the advantage to allow the selection for orientation-specific or not whereas PRO-seq is inherent and obligatory to PRO-seq.

Thank you, we have rephrased the advantage list to more accurately describe this point and better acknowledge the strength of RNA-seq. What we meant is the advantage is that strand specificity is captured by default in PRO-seq, whereas in RNA-seq, strand-specificity is not captured unless specifically applied. We do not consider it an advantage to be able to ignore strand specificity. The text now reads, “nascent RNA profiling measures not only RNA polymerase occupancy, but also orientation by default, whereas traditional RNA-seq requires specific library preparation steps to capture orientation”.

3) My previous question #6 related with the normalization in Figs. 2B, 8C and S1 is answered as “we have updated plots” but eh 2B & 8C plots are identical to the previous version. In the legend it is said they are normalized. Therefore, one can conclude that they were already normalized in the previous version. If that is true why the authors say the they updated the graphs? If not, why they show an identical plot to the previous unnormalized version?

We apologize for the confusion here. We have gone back and double-checked everything and can now explain. In the revised 2B and 8C, the plots are, in fact, updated; but you’re right, they are also identical to the original plots. The reason this happened is that we scaled each sample individually. So, since we removed the scale bars, and since the scales are free, the result was identical to when they were unnormalized. So, we did in fact update the plots, but they didn’t change; hence the confusion. We realize now you may have wanted them to be on a fixed scale, rather than free scale, so we tried that (see figure R1). Unfortunately, the problem with the fixed scale is that these tracks have very different levels in the region we’re showing. In the end, we find the point illustrated best in with free scales, so we’ve left that figure in the paper.

Regarding S1, the very nature of the comparison is to illustrate how increasing the number

of reads derived from RNA-seq increases the coverage at exonic regions. Were this to be normalized by read count, the exercise to be illustrated would be masked by the nature of the normalization. Therefore, these tracks were not normalized.

Fig. R1: **Fixed-scale tracks**. When using fixed scales these plots aren’t useful because of the very different signal levels at different samples, even after normalization. Hence, we elected to use free scale tracks in the publication.

4) My previous question #12 for the use of rDNA abundance as a control of mature RNA is not convincingly answered. The plot shown in Fig. R3 left shows a good correlation between rDNA level and mRNA contamination. However, the right graph shows that this good correlation strongly depends on the HeLaS3 point. The R goes bak to 0.48 and, importantly, the p-value is NOT significant (0.11)

Thank you for bringing up this point again, it caused us to go back and double-check this point. You’re right about the figure; originally, our primary goal there was to illustrate that they both independently identify HeLaS3 as a problematic library. But to dig into this deeper, we went back and included an additional analysis, adding all of the non-redundant samples from GSE126919, which incorporates samples derived from three separate clones, in that plot. With the additional number of samples, our revised plot more convincingly illustrates a significant correlation between rRNA alignment and mRNA contamination even in the absence of the outlier HelaS3 sample. We think this new plot makes an important point, and more fully addresses your original concern, so we added it in the paper, in Figure S4 (reproduced here as figure R2). We also revised the section of text explaining this. Even though the corelation is lower, there’s a significant relationships. We interpret this as meaning that these two measures are not exactly the same, but they are related – so we view it as beneficial to include both as output in PEPPRO.

Fig. R2: **Abundance of rDNA to total reads is correlated with mature RNA contamination.** Correlation plot between the measure of mRNA contamination (median exon:intron density) and the ratio of rDNA aligned reads to total reads for: A) all primary samples (*excludes RNA-seq spike-in experiment due to ribosomal depletion inherent in RNA-seq library preparation), B) primary samples excluding the known outlier HelaS3 GRO-seq sample, C) all samples in panel B and all non-redundant samples from GSE126919 including three cellular subclones (A, B, and C) to demonstrate possible differences due to cell lines. Test for association determined with Pearson’s product moment correlation coefficient.

**3^rd^ round**

**Reviewer 2**

All the answers from the authors are satisfactory but the point number 3 where the authors say that "--illustrated best in free scales". In Figures 2B and 8C is not possible to compare between samples if the range of the free scale is not given. I agree that free scales are necessary to see samples with large differences in intensity but the reader should have the opportunity to compare the scales used in each sample.
